# Arsenic Handicap? Prenatal Exposure Worsens Influenza Infections in Young Mice

**DOI:** 10.1289/ehp.121-A312

**Published:** 2013-10-01

**Authors:** Carol Potera

**Affiliations:** Carol Potera, based in Montana, has written for *EHP* since 1996. She also writes for *Microbe*, *Genetic Engineering News*, and the *American Journal of Nursing*.

Research in adult animals has provided evidence that exposure to inorganic arsenic may compromise immune responses to bacterial and viral infections.^1^ Arsenic exposure has also been associated with altered gene expression in lung tissue from mice, including reduced expression of genes involved in innate immune responses.^2^ But it’s still unclear how arsenic might modify acute inflammatory responses during early-life infections and the subsequent effects on lung structure and function.

Now Kathryn Ramsey, a respiratory physiologist at the Telethon Institute for Child Health Research in Subiaco, Western Australia, and her colleagues show that mice exposed to inorganic arsenic *in utero* and infected with influenza A soon after birth did not fight off the viral infection as well as unexposed mice. The arsenic-exposed mice also had more lung inflammation and airway damage. These findings, reported in this issue of *EHP*, strengthen the connection between early arsenic exposure and the development of chronic lung problems.^3^

Arsenic is a common contaminant of drinking water worldwide, and high exposures to the ubiquitous toxicant are known to increase the risk of lung, skin, bladder, and other cancers.^4^ The U.S. Environmental Protection Agency (EPA) has set a maximum contaminant level of 10 ppb for total arsenic in public drinking water supplies—that includes both inorganic and the less toxic organic forms of arsenic.^5^ Foods including rice, rice-based products, and apple juice also can be contaminated with inorganic arsenic, and diet may constitute a sizeable proportion of some people’s total exposure.^6^ Arsenic is not currently regulated in food, although the U. S. Food and Drug Administration recently proposed guidance for industry that levels of inorganic arsenic in apple juice not to exceed 10 ppb.^7^

In addition to cancer, inorganic arsenic has been linked to nonmalignant respiratory illnesses including chronic bronchitis, chronic obstructive pulmonary disease, and bronchiectasis in people, with lung cancer and bronchiectasis particularly linked to exposure *in utero* or during early childhood.^8^ Bronchiectasis is a progressive respiratory disease caused by repeated lower respiratory tract infections and exaggerated inflammatory responses to respiratory pathogens, which can lead to serious heart and lung problems.

**Figure 1:**
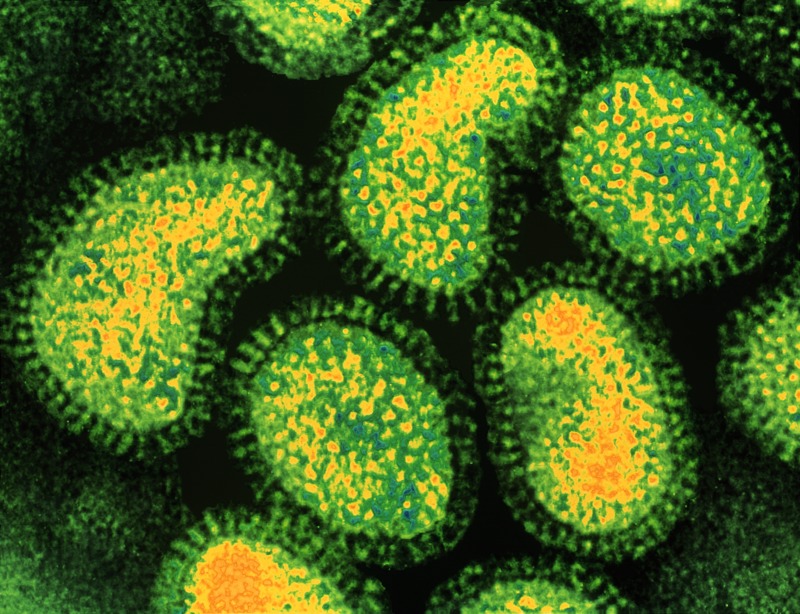
Colored transmission electron micrograph of influenza A viruses. Each virus is made up of an RNA core (yellow) surrounded by a protein envelope (green). The spiked surface of the envelope enables the virus to stick to host cells. © Dr. Linda M. Stannard, University of Cape Town/Science Source

In the current study, pregnant C57BL/6 mice drank either clean water or water contaminated with 100 ppb inorganic arsenic.^3^ One week after birth, some pups were intranasally inoculated with the H3N1 strain of influenza A. Pups of arsenic-exposed dams drank arsenic-treated water, and pups of unexposed dams drank clean water.^3^

One week after influenza infection, young mice exposed to both arsenic and influenza had viral titers and inflammatory responses that were 7–8 times higher than controls exposed to clean water and no influenza. At 8 weeks of age, the investigators measured cytokine levels in bronchoalveolar fluid to evaluate inflammatory responses and airway responsiveness to methacholine (a bronchoconstrictor). Mice exposed to both arsenic and influenza had significantly more airway resistance and greater deficits in lung mechanics than control mice.^3^

The effects of arsenic on immune function are unlikely to be relevant only to influenza, although the degree of lung damage may vary with other infections. For example, Ramsey says, exposure to different viruses may result in milder pathology. “We would like to combine arsenic exposure with repeated bacterial and viral infections to model the development of bronchiectasis throughout life,” she says.

“This is a great study that advances the field,” says Bruce Stanton, director of the Toxic Metals Superfund Research Program at Dartmouth College’s Geisel School of Medicine. He says the results suggest that infants and young children exposed to inorganic arsenic may be more susceptible to influenza infections and subsequent lung damage.

Although the 100-ppb arsenic exposure is relevant to water levels in some parts of the world, “it’s on the high side for the United States, where the EPA limit is 10 ppb,” says Stanton. However, the EPA standard does not cover private wells; by one estimate, as many as 25 million people in the United States who obtain their drinking water from private wells may be exposed to arsenic levels above the EPA standard.^1^

Stanton suggests repeating the mouse experiments with 10 ppb inorganic arsenic to see if the influenza response still worsens. Given current guidance on arsenic in drinking water and apple juice, “We’d want to know that,” says Stanton.
